# Effectiveness and Types of Interventions for Autism Spectrum Disorder: A Systematic Review, Meta-Analysis, and Meta-Regression

**DOI:** 10.7759/cureus.111009

**Published:** 2026-06-17

**Authors:** John K Muthuka, Ruvimbo Zimunya, Andrina Simengwa, Chrisphine Onyango, Kelly Oluoch, Mary T Kioko, Dianna F Mbari, Japheth Nzioki, Lucy K Chebungei, Sara Kim, Desire Desire Nshimirimana

**Affiliations:** 1 Epidemiology, Public Health and Biostatistics, Jomo Kenyatta University of Agriculture and Technology, Nairobi, KEN; 2 Public Health Sciences, Kenya Medical Training College, Nairobi, KEN; 3 Children Behavior Services, Anderson Center for Autism, New York, USA; 4 Pharmacy, Kenya Medical Training College, Nairobi, KEN; 5 Nursing, Kenya Medical Training College, Nairobi, KEN; 6 Public Health, Alberta Health Services, Edmonton, CAN; 7 School of Nursing, Andrews University, Berrien Springs, USA; 8 College of Doctoral Studies, Grand Canyon University, Phoenix, USA

**Keywords:** autism spectrum disorder, bayesian meta-analysis, behavioral interventions, meta-analysis, systematic review

## Abstract

This systematic review and meta-analysis aimed to estimate the overall effectiveness of autism spectrum disorder (ASD) interventions and identify sources of heterogeneity using frequentist and Bayesian approaches. A systematic search of PubMed/MEDLINE, Embase, Web of Science, and Scopus was conducted for studies published between January 1, 2004, and April 30, 2025. Primarily, randomized controlled trials with extractable intervention outcomes were included. A total of 41 studies (n = 3,008) were synthesized using random-effects models (restricted maximum-likelihood (REML)), Bayesian hierarchical modeling, meta-regression, and sensitivity analyses following PRISMA guidelines. The pooled random-effects estimate showed a significant positive effect of ASD interventions (effect size = 0.506, 95% CI: 0.392-0.619; z = 8.72, p < 0.001), corresponding to an estimated success proportion of 62% (95% CI: 59%-65%). Heterogeneity was substantial (Qₑ (40) = 238.78, p < 0.001; I² = 82.45%; τ² = 0.069, 95% CI: 0.028-0.137; τ = 0.262), with H² = 5.70 and a wide prediction interval (-0.020 to 1.031), indicating strong between-study variability. Bayesian meta-analysis confirmed a comparable effect (posterior mean = 0.619 (62%), 95% CrI: 0.592-0.646), with τ = 0.273 and I² ≈ 82.5%; Markov Chain Monte Carlo (MCMC) diagnostics indicated stable convergence (R-hat ≈ 1.00). Publication bias analyses indicated significant funnel plot asymmetry (Egger-type regression: z = 3.429, p < 0.001; weighted regression: t = 9.573, p < 0.001), while rank correlation was non-significant (τ = −0.178, p = 0.103). Trim-and-fill analysis imputed 10 studies, reducing the pooled effect to 0.374 (37%; 95% CI: 0.258-0.491; τ = 0.338), although the effect remained significant (p < 0.001). Sensitivity analyses excluding influential studies yielded a stable effect (0.505 (51%), 95% CI: 0.401-0.609), with persistent heterogeneity (I² = 75.49%; Qₑ (38) = 190.21, p < 0.001; τ² = 0.043). Subgroup analyses showed highest effects for digital/technology-based interventions (0.672 (67%); I² = 0%), followed by nutritional (0.635 (64%); I² = 73.81%), behavioral (0.630 (63%); I² = 74.78%), and pharmacological (0.627 (63%); I² = 0%) interventions, while physical/occupational therapies showed lower effects (0.523 (52%); I² = 63.35%) and combined interventions showed borderline effects (0.593 (59%); I² = 19.96%); subgroup differences were significant (Q(5) = 22.63, p < 0.001). Regional effects were similar and non-significant across North America, Europe, and Asia. Meta-regression identified significant moderators including intervention context (Qₘ = 18.159, p = 0.020), outcome domain (Qₘ = 19.588, p = 0.003), age at intervention onset (Qₘ = 17.795, p = 0.003), and intervention category (Qₘ = 31.714, p < 0.001), while follow-up and intervention duration were not significant. Bayesian subgroup analyses confirmed strongest evidence for pharmacological, behavioral, and digital interventions. Overall, ASD interventions demonstrated a moderate and statistically significant overall effect (~0.50-0.62 (50-62%)), with substantial heterogeneity driven primarily by intervention type, context, and participant characteristics. Findings were consistent across frequentist, Bayesian, and sensitivity analyses, supporting robust but context-dependent effectiveness.

## Introduction and background

Autism spectrum disorder (ASD) is a neurodevelopmental condition characterized by persistent differences in social communication and restricted, repetitive patterns of behavior that typically emerge in early childhood [1-5]. The global burden of ASD has become an increasing public health priority due to its lifelong functional implications and substantial social and economic costs, with recent estimates indicating that ASD affects approximately 1 in 100 children worldwide, underscoring its growing public health significance and the need for effective interventions and support systems [6]. While accurate prevalence estimates are essential for informing health policy and guiding early detection strategies, equally critical is the evaluation of intervention effectiveness, given the need to improve developmental outcomes and reduce disability across diverse populations. This systematic review and meta-analysis were conducted in accordance with PRISMA 2020 guidelines.

Over the past two decades, a wide range of interventions - including behavioral, developmental, educational, and pharmacological approaches - have been implemented worldwide. Evidence from randomized controlled trials (RCTs) and systematic reviews suggests that structured behavioral interventions, such as applied behavior analysis (ABA) and naturalistic developmental behavioral interventions (NDBI), consistently improve communication, adaptive functioning, and cognitive skills [7,8]. Broader early childhood programs and comprehensive developmental interventions have also demonstrated benefits, though their effects are more variable and context-dependent [9]. Despite these advances, substantial heterogeneity in reported outcomes persists, reflecting differences in study design, intervention intensity, participant characteristics, and healthcare infrastructure.

Previous meta-analyses have provided pooled estimates of intervention effectiveness, but many have focused narrowly on specific modalities or regions, leaving gaps in understanding the broader drivers of variability. Given ongoing changes in diagnostic practices, awareness, and intervention delivery from 2004 to 2025, an updated synthesis is needed to quantify both the overall effectiveness of autism interventions and the methodological and contextual moderators contributing to heterogeneity.

The primary objective of this study was to evaluate the overall effectiveness of interventions for ASD across diverse populations, while the secondary objectives were to assess intervention-specific effectiveness and identify sources of between-study heterogeneity using Bayesian meta-analysis and meta-regression techniques. This dual focus provides not only a precise estimate of intervention impact but also critical insights into the conditions under which interventions are most effective, thereby informing clinical practice, policy development, and future research.

## Review

Materials and methods

Study Design and Protocol Registration

A systematicreview and meta-analysis of ASD intervention studies was carried out. The study adhered to the Preferred Reporting Items for Systematic Reviews and Meta-Analyses guidelines [10,11]. Reporting followed PRISMA 2020 guidelines for systematic reviews and meta-analyses. The current review is part of a larger, prospectively registered study titled Global Trends in Autistic Disorder Diagnosis and Intervention Outcomes-A Systematic Review, Meta-analysis, and Meta-Regression (PROSPERO: CRD420251069271), with a particular emphasis on evaluating the effect of the ASD interventions cumulatively and also, specifically as per the different regions of the globe, spanning 2004 to 2025. This review specifically included RCTs only.

Search Strategy

A comprehensive electronic search was conducted in PubMed/MEDLINE, Embase, Web of Science, and Scopus for studies published between January 1, 2004, and April 30, 2025. Grey literature sources were also searched to identify additional relevant studies. The search strategy combined controlled vocabulary terms, including Medical Subject Headings (MeSH), with free-text keywords such as “autism spectrum disorder,” “intervention,” “therapy,” “treatment,” “behavioral,” “pharmacological,” “technology-based,” and “developmental,” using Boolean operators (AND/OR). No language or geographic restrictions were applied. Search terms were structured according to the PICO framework to ensure comprehensive coverage of ASD intervention studies. The population component included individuals diagnosed with ASD across all age groups, settings, and diagnostic criteria. Interventions encompassed any therapeutic approach targeting ASD, including behavioral interventions (e.g., ABA and NDBI), developmental and educational programs, digital or technology-based interventions, nutritional therapies, physical and occupational therapies, and pharmacological treatments. Comparators included standard care, wait-list controls, placebo, or alternative intervention arms reported in RCTs. Outcomes focused on quantitative measures of intervention effectiveness, including communication, adaptive functioning, cognitive outcomes, behavioral symptoms, social interaction, and global improvement scores. Only RCTs with extractable quantitative outcomes were eligible for inclusion. These PICO elements guided the development of database-specific search strings and ensured that all studies meeting the predefined eligibility criteria were systematically captured (see Appendix A).

Study Selection

Studies were included if they were RCTs, evaluated interventions targeting individuals with ASD, and reported extractable quantitative outcomes on intervention effectiveness. Study selection followed PRISMA 2020 screening and eligibility procedures, including title and abstract screening followed by full-text review. All retrieved records were screened independently by two reviewers at both the title/abstract and full-text stages. Studies meeting the predefined eligibility criteria were included in the review. Discrepancies between reviewers were resolved through discussion and consensus, with consultation from a third reviewer when required. Inter-reviewer agreement statistics were not formally calculated.

Eligibility Criteria

This systematic review and meta-analysis included RCT studies documenting the type of ASD intervention alongside its effect on ASD and were conducted in any part across the globe. Studies were excluded if they were experimental, case series, case reports, letters, opinions, narrative reviews, or other studies that did not contain primary data, if they were duplicates, or if they did not have full text. Studies lacking clear diagnostic criteria or denominators were excluded from quantitative synthesis.

Data Extraction

In case of duplicate publications, the study with the most complete dataset was included. Extracted variables included publication year, country, study design, ASD diagnostic tool, intervention type and category, intervention intensity, participant age, sample size, outcome domain, follow-up duration, and study context (e.g., clinical or educational). Because the included studies reported intervention effects using different outcome measures and scales, effect sizes were converted to standardized mean differences (Hedges' g) with corresponding standard errors prior to meta-analysis. This standardization enabled the pooling of outcomes across diverse domains, including social communication, adaptive functioning, cognition, behavioral symptoms, sleep, and overall developmental outcomes, by expressing intervention effects on a common scale.

Risk of Bias of Assessment

Risk of bias was assessed using a domain-based approach appropriate for RCTs and visualized using the Risk-of-Bias Visualization (ROBVIS) tool [12]. Domains included randomization process, deviations from intended interventions, missing outcome data, outcome measurement, and selective reporting. Each study was rated as low risk, some concerns, or high risk of bias. Two reviewers independently conducted assessments, with disagreements resolved through consensus or a third reviewer.

Data Synthesis and Statistical Analysis

Non-meta-analyzed study characteristics were narratively described, including author, year, country, design, and sample size. Effect sizes for ASD interventions were extracted and pooled where possible. Meta-analyses were conducted using JASP (Version 0.95.4.0; JASP Team, Amsterdam, the Netherlands) [13-15] with random-effects models estimated via restricted maximum-likelihood (REML). Effect sizes were logit-transformed and back-transformed for interpretability. Heterogeneity was assessed using Cochran’s Q, I², and τ² statistics. Influence diagnostics and Baujat plots were used to identify outlier studies. Funnel plots and residual funnel plots were used to assess small-study effects.

Because the included studies evaluated ASD interventions across multiple but conceptually related outcome domains, effect sizes were transformed to a common metric to enable synthesis while accounting for differences in measurement instruments. A random-effects model was used to accommodate anticipated between-study heterogeneity arising from variations in interventions, participants, settings, and study designs. For analyses involving pooled proportions, study-specific proportions were logit-transformed prior to meta-analysis to stabilize variances and improve statistical performance, particularly for proportions near 0 or 1. Pooled estimates were subsequently back-transformed to the original proportion scale to facilitate interpretation.

Meta-Regression Analysis

Meta-regression analyses examined pre-specified moderators including intervention type, context, follow-up duration, outcome domain, age at intervention onset, and intervention duration. Omnibus tests were followed by coefficient-level interpretation. Multi-collinearity was assessed using variance inflation factors (VIFs) [16], which informed model interpretation and refinement.

Sensitivity and Robustness Analysis

Model stability was further evaluated through sensitivity analyses and influence diagnostics. These included leave-one-out analyses, exclusion of influential studies, and comparison of model estimates after removing studies contributing to sparse or unstable category estimates, particularly within multi-level categorical moderators. These procedures were used to assess the robustness of both the pooled effect size and the observed moderator effects.

Bayesian Meta-Analysis

Bayesian random-effects meta-analysis [17,18] was performed using weakly informative priors, with a Normal (0,1) prior specified for the pooled logit effect size and a Half-Cauchy (0,1) prior for between-study heterogeneity (τ). Markov Chain Monte Carlo (MCMC) sampling [19-23] was conducted with appropriate burn-in and iteration settings. Convergence was assessed using R-hat statistics and effective sample sizes. Posterior distributions were examined to evaluate the precision of effect estimates and to confirm that estimates were well-separated from the null, indicating robust evidence of intervention effects.

Results

Included Articles and Quality Assessment (Systematic Review)

**Figure 1:**
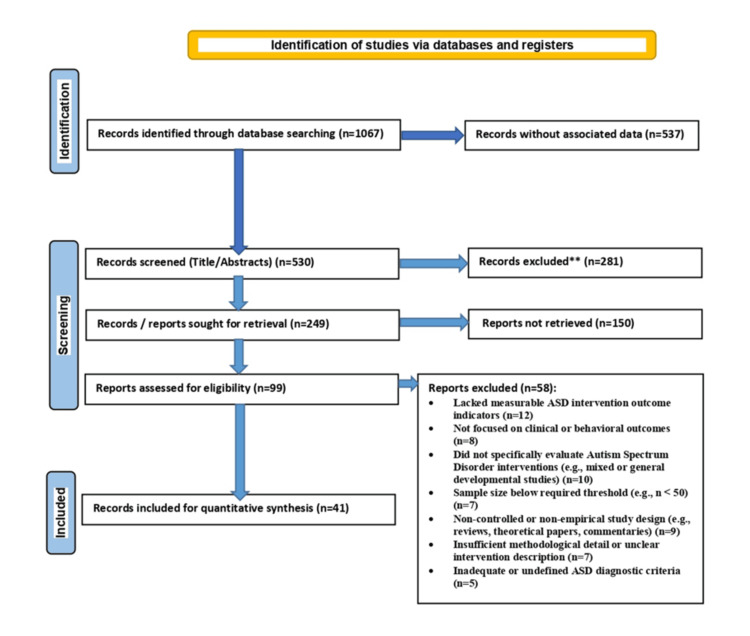
Flow diagram of study identification and selection according to the Preferred Reporting Items for Systematic Reviews and Meta-Analyses (PRISMA) guidelines. **Records excluded after title/abstract screening due to not meeting inclusion criteria (wrong population, intervention, outcome, or study design). ASD: autism spectrum disorder

The database search identified 1,067 records. After removing 537 articles without associated data as described, 530 records remained for title and abstract screening. Of these, 281 were excluded, and 249 reports were sought for retrieval. A total of 150 reports could not be retrieved, leaving 99 full-text articles assessed for eligibility. After full-text evaluation, 58 articles were excluded for predefined reasons. Ultimately, 41 studies [24-64] met the inclusion criteria and were included in the quantitative synthesis (Figure 1).

Features of the Included Studies

Forty-one included studies [24-64] (n = 3,008) evaluated a broad range of ASD interventions, predominantly behavioral and psychosocial approaches such as parent training, cognitive behavioral therapy (CBT), social skills, peer-mediated, caregiver-mediated, theatre-based, psychoeducational, and positive behavior support interventions [25,32-36,38,40,41,44,49,53,56,57,60,61,63]. Additional studies examined pharmacological interventions [26,28,37,39,43,47,52,55,58,64], nutritional and biomedical approaches [24,42,45,50,51], physical and occupational therapies [31,48,59], digital and technology-based interventions [29,30,54], and combined multimodal approaches [46,62]. Most studies were RCTs conducted in North America and Europe and primarily involved children with ASD. Interventions were commonly short-term and delivered across home, clinic, school, and community settings. Outcomes mainly included ASD symptoms, social communication, behavior, sleep, adaptive functioning, and family functioning, with follow-up typically limited to post-treatment assessment. Overall effects were generally moderate, although stronger evidence was observed for several behavioral and psychosocial interventions (Table 1).

**Table 1:** Features of the studies included in the meta-analysis. Duration categories were classified as short (<3 months), medium (3-6 months), and long (>6 months). Intensity categories were classified as low (<weekly or limited exposure), high (≥2× per week or ongoing), or daily/continuous dosing. Adams et al. [24] combined dietary modification with vitamin, mineral, and fatty acid supplementation. Corbett et al. [61,63] evaluated theatre-based interventions targeting social competence in autistic youth and adults, while Fatta et al. [57] examined the PEERS® structured social skills program. Green et al. [34,35] focused on infants at elevated familial likelihood for ASD using parent-mediated interventions, and Guzick et al. [36] delivered parent-led CBT online through email or telehealth support. Malow et al. [64] assessed long-term melatonin treatment for sleep and growth outcomes, whereas Minshawi et al. [46] combined D-cycloserine pharmacotherapy with behavioral social skills training. Randell et al. [48] investigated Sensory Integration Therapy for sensory processing difficulties, and Strydom et al. [53] included adults with co-occurring intellectual disability and ASD. McKenzie et al. [60] was rated as “Not suitable” because no applicable post-treatment effectiveness outcome was available. ASD: autism spectrum disorder; ADHD: attention-deficit/hyperactivity disorder; CBT: cognitive behavioral therapy; GI: gastrointestinal; PEERS®: Program for the Education and Enrichment of Relational Skills; PACT: Paediatric Autism Communication Therapy; PBS: Positive Behaviour Support; TBW: Therapeutic Body Wraps; ID: intellectual disability

Study ID	Sample Size	Continent	Intervention Category	Intervention Type	Duration	Intensity	Age at Start	Population	Outcome Domains	Follow-Up	Retention	Context	Effectiveness
Adams et al. [24]	117	North America	Nutritional and Biomedical	Comprehensive nutritional and dietary intervention (vitamins, minerals, fatty acids, diet changes)	Long (>6 months)	High (≥2× per week/ongoing)	Children	ASD	Core ASD symptoms; adaptive behavior; cognition; GI symptoms	12 months	Reported	Home-based	Moderate
Bearss et al. [25]	180	North America	Behavioral and Psychosocial	Parent training program	Medium (3-6 months)	Low (< weekly/limited exposure)	Children	ASD + disruptive behavior	Behavioral problems; adaptive behavior	Post-treatment	High	Clinic-based	Moderate
Chugani et al. [26]	111	North America	Pharmacological	Low-dose buspirone	Medium (3-6 months)	High (≥2× per week/ongoing)	Children	ASD	Restricted and repetitive behaviors	Post-treatment	Reported	Clinic-based	Moderate
Corbett et al. [61]	77	North America	Behavioral and Psychosocial	Theatre-based intervention	Short (<3 months)	Low (< weekly/limited exposure)	Youth	ASD	Social cognition; social behavior	Post-treatment	Reported	Community-based	Moderate
Corbett et al. [63]	47	North America	Behavioral and Psychosocial	Theatre-based intervention (enhanced for adults)	Short (<3 months)	Low (< weekly/limited exposure)	Adults	ASD	Social competence	Post-treatment	Reported	Community-based	Moderate
Delion et al. [59]	41	Europe	Physical and Occupational Therapies	Therapeutic Body Wraps (TBW)	Short (<3 months)	High (≥2× per week/ongoing)	Children	ASD with severe injurious behavior	Injurious behavior; agitation	Post-treatment	Reported	Inpatient/clinical	Moderate
DeVane et al. [28]	61	North America	Pharmacological	Multi-drug pharmacotherapy (BAART trial)	Short (<3 months)	High (≥2× per week/ongoing)	Children	ASD	Behavioral symptoms; global functioning	Post-treatment	Reported	Clinic-based	Moderate
Fatta et al. [57]	37	Europe	Behavioral and Psychosocial	PEERS® social skills program	Medium (3-6 months)	Low (< weekly/limited exposure)	Children and Adolescents	Autistic adolescents	Social skills; social functioning	Post-treatment	Reported	Clinic-based	Moderate
Fletcher-Watson et al. [29]	49	Europe	Digital and Technology-Based	iPad-based social communication intervention	Short (<3 months)	High (≥2× per week/ongoing)	Children	ASD	Social communication	Post-treatment	Reported	Home-based	Moderate
Frazier et al. [30]	90	North America	Digital and Technology-Based	Sound-to-sleep mattress technology	Short (<3 months)	High (≥2× per week/ongoing)	Children	ASD + sleep difficulties	Sleep outcomes	Immediate	Complete	Home-based	Limited
Gabriels et al. [31]	116	North America	Physical and Occupational Therapies	Therapeutic horseback riding	Short (<3 months)	Low (< weekly/limited exposure)	Children and Adolescents	ASD	Social functioning; behavior	Post-treatment	Reported	Community-based	Moderate
Gordon et al. [32]	48	Europe	Behavioral and Psychosocial	PEGASUS psychoeducational program	Short (<3 months)	High (≥2× per week/ongoing)	Youth	High-functioning ASD	Self-awareness; psychosocial adjustment	Post-treatment	Reported	Clinic-based	Moderate
Green et al. [33]	152	Europe	Behavioral and Psychosocial	PACT (parent-mediated communication intervention)	Long (>6 months)	High (≥2× per week/ongoing)	Children	ASD	Social communication; autism symptoms	Long-term	High	Home-based	Strong
Green et al. [35]	54	Europe	Behavioral and Psychosocial	Parent-mediated early intervention	Medium (3-6 months)	High (≥2× per week/ongoing)	Infants	High-risk for ASD	Social communication; early ASD markers	Post-treatment	Reported	Home-based	Moderate
Green et al. [34]	54	Europe	Behavioral and Psychosocial	Parent-mediated intervention (follow-up of early trial)	Long (>6 months)	High (≥2× per week/ongoing)	Infants	High-risk for ASD	Developmental outcomes; ASD symptoms	3 years	Reported	Home-based	Strong
Guzick, et al. [36]	57	North America	Behavioral and Psychosocial	Internet-based parent-led CBT (email vs telehealth support)	Short (<3 months)	Low (< weekly/limited exposure)	Youth	ASD + anxiety disorders	Anxiety symptoms; functioning	Post-treatment	Reported	Home-based	Moderate
Hendren et al. [37]	57	North America	Pharmacological	Methyl B12 supplementation	Short (<3 months)	High (≥2× per week/ongoing)	Children	ASD	Autism symptoms; communication	Post-treatment	Reported	Clinic-based	Moderate
Ibañez et al. [38]	104	North America	Behavioral and Psychosocial	Web-based tutorial for parents	Short (<3 months)	Low (< weekly/limited exposure)	Children	ASD	Parent-child interactions; ASD symptoms	Post-treatment	Reported	Home-based	Moderate
Ichikawa et al. [39]	92	Asia	Pharmacological	Aripiprazole for irritability	Short (<3 months)	High (≥2× per week/ongoing)	Children and Adolescents	ASD	Irritability; behavior	Post-treatment	Reported	Clinic-based	Moderate
Kamps et al. [40]	95	North America	Behavioral and Psychosocial	Peer network intervention	Short (<3 months)	Low (< weekly/limited exposure)	Children	ASD	Social communication; peer interactions	Post-treatment	Reported	School-based	Strong
Kasari et al. [41]	86	North America	Behavioral and Psychosocial	Parent-mediated intervention	Medium (3-6 months)	High (≥2× per week/ongoing)	Toddlers	ASD	Communication skills; social behavior	Post-treatment	Reported	Home-based	Strong
Keim et al. [42]	21	North America	Nutritional and Biomedical	Omega-3 and Omega-6 fatty acids	Short (<3 months)	High (≥2× per week/ongoing)	Children	ASD	Inflammatory markers; behavioral symptoms	Post-treatment	Reported	Clinic-based	Moderate
Lemonnier et al. [43]	60	Europe	Pharmacological	Bumetanide (diuretic)	Short (<3 months)	Low (< weekly/limited exposure)	Children	ASD	Autism symptoms; irritability	Post-treatment	Reported	Clinic-based	Moderate
Lopata et al. [27]	90	North America	Behavioral and Psychosocial	Social intervention for children	Medium (3-6 months)	High (≥2× per week/ongoing)	Children	ASD	Social skills; peer interactions	Long-term	Reported	School-based	Strong
Malow et al. [64]	95	North America	Pharmacological	Prolonged-release melatonin	Long (>6 months)	High (≥2× per week/ongoing)	Children	ASD	Sleep patterns; growth	Long-term	High	Home-based	Strong
Marshall et al. [44]	50	Europe	Behavioral and Psychosocial	Social Stories intervention	Short (<3 months)	High (≥2× per week/ongoing)	Children	ASD in mainstream schools	Social behavior; classroom functioning	Post-treatment	Reported	School-based	Moderate
McKenzie et al. [60]	34	Europe	Behavioral and Psychosocial	SAFE family-based intervention	Short (<3 months)	High (≥2× per week/ongoing)	Children	ASD	Family functioning; stress	Not applicable	Not applicable	Clinic-based	Not suitable
Mehrazad-Saber et al. [45]	43	Asia	Nutritional and Biomedical	L-carnosine supplementation	Short (<3 months)	High (≥2× per week/ongoing)	Children	ASD	Sleep disorders; ASD severity	Post-treatment	Reported	Clinic-based	Moderate
Minshawi et al. [46]	68	North America	Combined (Pharmacological + Behavioral)	D-cycloserine + social skills training	Short (<3 months)	Low (< weekly/limited exposure)	Children and Adolescents	ASD	Social skills	Post-treatment	Reported	Clinic-based	Moderate
Quintana et al. [47]	32	Europe	Pharmacological	Intranasal oxytocin (Breath Powered device)	Short (<3 months)	High (≥2× per week/ongoing)	Adults	ASD	Social cognition; social behavior	Immediate	Reported	Clinic-based	Moderate
Randell et al. [48]	138	Europe	Physical and Occupational Therapies	Sensory Integration Therapy	Short (<3 months)	High (≥2× per week/ongoing)	Children	ASD with sensory processing difficulties	Sensory processing, adaptive behavior	Post-treatment	Reported	Clinic-based	Moderate
Roberts et al. [49]	93	North America	Behavioral and Psychosocial	Caregiver-mediated social communication training	Short (<3 months)	High (≥2× per week/ongoing)	Toddlers	ASD	Social communication	Post-treatment	Reported	Clinic and home	Strong
Rojo-Marticella et al. [50]	42	Europe	Nutritional and Biomedical	Probiotics	Short (<3 months)	High (≥2× per week/ongoing)	Children and Adolescents	ASD and/or ADHD	Behavioral, gastrointestinal	Post-treatment	Reported	Clinic-based	Moderate
Rossigno et al. [51]	62	North America	Nutritional and Biomedical	Hyperbaric treatment	Short (<3 months)	High (≥2× per week/ongoing)	Children	ASD	Behavior, social responsiveness	Post-treatment	Reported	Hospital-based	Moderate
Smith et al. [62]	56	North America	Combined (Pharmacological + Behavioral)	Atomoxetine + Parent Training	Medium (3-6 months)	High (≥2× per week/ongoing)	Children	ASD + ADHD	ADHD symptoms, adaptive behavior	Post-treatment	Reported	Clinic-based	Moderate
Strathearn et al. [52]	32	Oceania	Pharmacological	Intranasal Oxytocin	Short (<3 months)	High (≥2× per week/ongoing)	Children	ASD	Social attention, visual systemizing	Post-treatment	Reported	Clinic-based	Moderate
Strydom et al. [53]	113	Europe	Behavioral and Psychosocial	Positive Behaviour Support (PBS) training	Medium (3-6 months)	High (≥2× per week/ongoing)	Adults	ID + ASD + challenging behavior	Behavioral outcomes, cost-effectiveness	Post-treatment	Reported	Institutional	Strong
Voss et al. [54]	71	North America	Digital and Technology-Based	Wearable Digital Intervention	Short (<3 months)	High (≥2× per week/ongoing)	Children	ASD	Socialization, adaptive behavior	Post-treatment	Reported	Clinic and home	Moderate
Wink et al. [55]	31	North America	Pharmacological	N-acetylcysteine	Short (<3 months)	High (≥2× per week/ongoing)	Youth	ASD	Irritability, repetitive behaviors	Post-treatment	Reported	Clinic/home	Moderate
Wood et al. [56]	107	North America	Behavioral and Psychosocial	CBT for anxiety	Medium (3-6 months)	High (daily/ongoing)	Children	ASD	Anxiety symptoms	Post-treatment	Reported	Clinic	Strong
Zimmerman et al. [58]	45	North America	Pharmacological	Sulforaphane	Short (<3 months)	Daily oral dosing	Children	ASD	Multiple autism-related outcomes	Post-treatment	Reported	Clinic	Moderate

Risk of Bias in Included Studies

**Figure 2:**
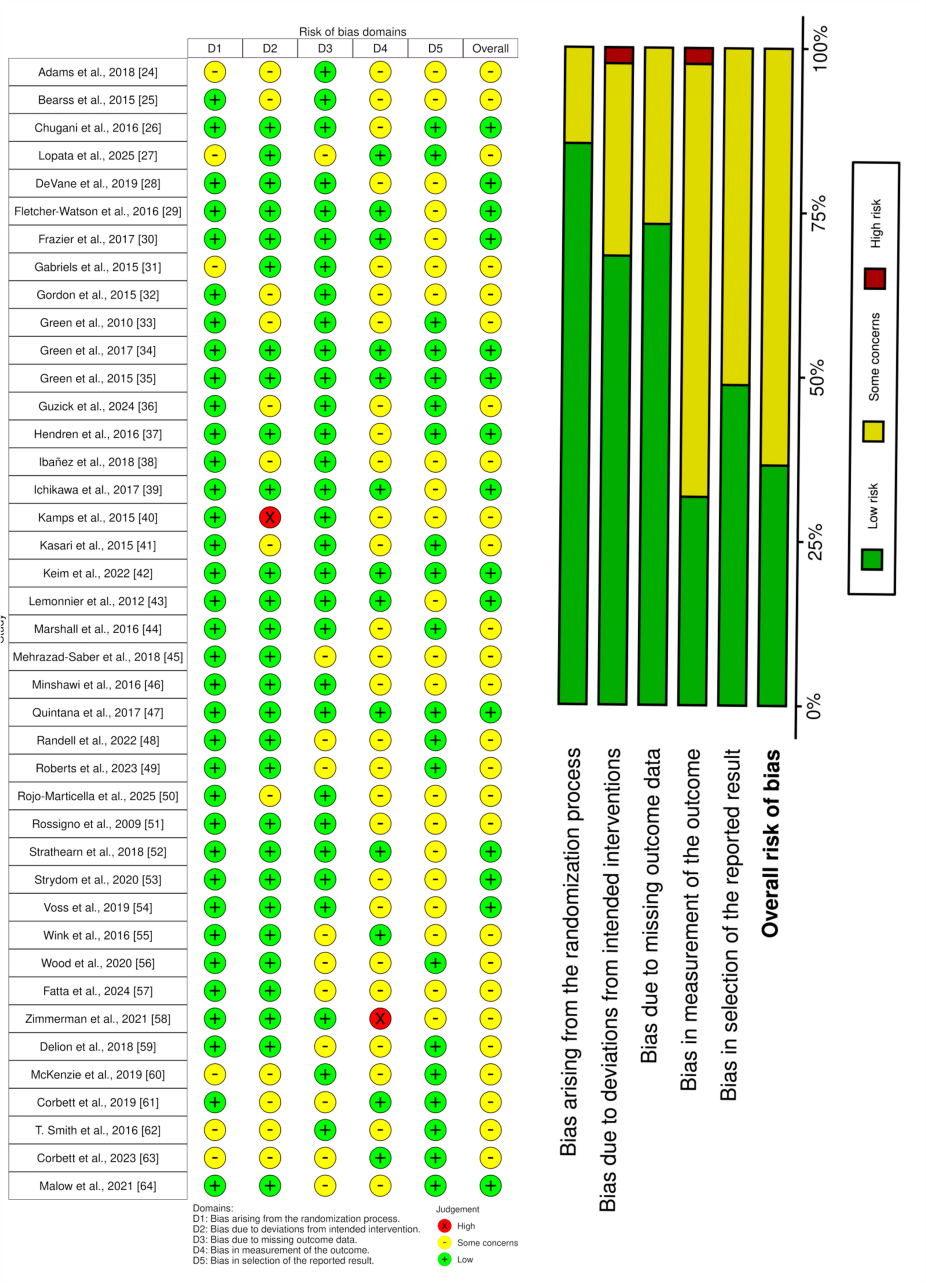
Risk of bias assessment of included studies using the Cochrane Risk of Bias 2 (RoB 2) tool and visualized using the ROBVIS tool. Studies included [24-64].

The overall risk of bias across the 41 included studies was moderate, with some variability across domains. Fifteen studies (37%) were rated as having low risk of bias, reflecting strong methodological rigor, including appropriate randomization, minimal missing data, and reliable outcome measurement. The remaining 26 studies (63%) were rated as having some concerns primarily due to deviations from intended interventions, limitations in outcome measurement, and potential selective reporting. Across domains, the randomization process was generally well conducted, and missing outcome data were largely well managed. In contrast, deviations from intended interventions and outcome measurement were the main sources of bias. Overall, the evidence base indicates a predominantly moderate risk of bias, with generally consistent findings suggesting beneficial intervention effects, although the certainty of evidence remains moderate (Figure 2 and Appendix B).

Meta-Analysis of Pooled Effect and Heterogeneity of the Interventions

**Figure 3:**
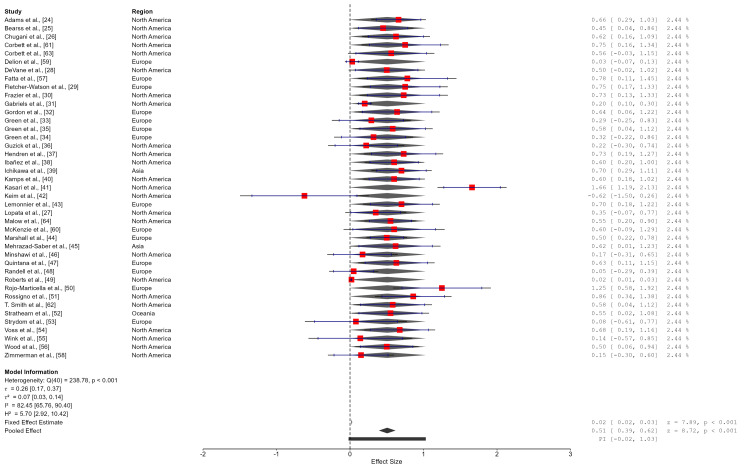
Forest plot of pooled effect of ASD interventions. Forest plot showing a statistically significant pooled effect of ASD interventions across studies (n=41) [24-64], based on a random-effects meta-analysis (effect size = 0.506, 95% CI (0.392, 0.619), p < 0.001). Substantial heterogeneity was observed (I² = 82.45%), indicating variability in effects across studies. ASD: autism spectrum disorder Studies included [24-64].

A random-effects meta-analysis was conducted to estimate the overall effect of ASD interventions across diverse populations. The overall pooled effect was statistically significant, z = 8.72, p < 0.001. The pooled effect size was 0.506, 95% CI (0.392, 0.619), corresponding to an estimated proportion of 62% (95% CI (59%, 65%)) when back-transformed to the proportion scale. These results indicate that, on average, a substantial proportion of individuals benefit from the interventions across the populations studied. Heterogeneity among studies was substantial (Qₑ (40) = 238.78, p < 0.001), with a large between-study variance, τ² = 0.069, 95% CI (0.028, 0.137); τ = 0.262, 95% CI (0.167, 0.371). This indicates that the true effect sizes varied considerably across studies, likely due to differences in study populations, intervention types, or implementation contexts. Consistent with this, heterogeneity was high (I² = 82.45%), indicating that most of the observed variability reflects real differences rather than sampling error. The H² statistic further confirmed substantial dispersion (H² = 5.70, 95% CI (2.92, 10.42)), suggesting strong inconsistency across study-level effects. The 95% prediction interval ranged from -0.020 to 1.031 on the effect size scale, indicating that in some future contexts the intervention effect may be minimal or absent, while in others it may be very large. This wide prediction interval highlights the variability and contextual dependence of intervention effectiveness (Table 2 and Figure 3).

**Table 2:** Meta-analytic summary of ASD interventions. The pooled effect was transformed using a log-odds-to-proportions transformation. The back-transformed probability for the pooled effect indicates the estimated proportion of participants experiencing a positive outcome across interventions. 95% CI: 95% confidence interval; 95% PI: 95% prediction interval; τ: between-study standard deviation; τ²: between-study variance; Qₑ: Cochran’s Q for heterogeneity; I²: proportion of total variance due to heterogeneity; H²: relative excess heterogeneity; ASD: autism spectrum disorder

Parameter	Estimate	95% CI	95% PI
Pooled effect	0.506	0.392, 0.619	-0.020, 1.031
Between-study SD (τ)	0.262	0.167, 0.371	-
Between-study variance (τ²)	0.069	0.028, 0.137	-
I² (heterogeneity)	82.45%	65.76%, 90.40%	-
H² (heterogeneity)	5.70	2.92, 10.42	-

Bayesian Pooled Meta-Analysis Results and Diagnostics

**Figure 4:**
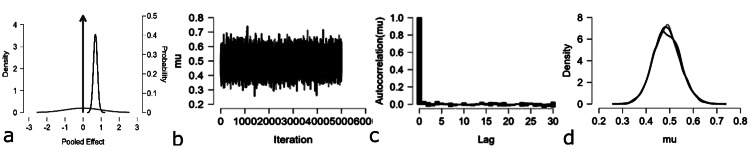
Markov Chain Monte Carlo (MCMC) diagnostic plots for the model parameters. (a) Posterior density of the pooled effect (μ), (b) trace plots across multiple chains, (c) autocorrelation function, and (d) posterior density of μ. The pooled effect is positive and precise (μ ≈ 0.62, 95% CI (0.593, 0.647)), although the prediction interval suggests variation across studies. The trace plots show well-mixed, stable chains, indicating good convergence. Autocorrelation decreases rapidly, showing efficient and mostly independent sampling. The posterior distribution is smooth and consistent across chains, while substantial heterogeneity (I² ≈ 82%) indicates notable differences between studies.

The Bayesian meta-analysis shows that autism interventions have a moderate-to-large positive effect, with a pooled mean of 0.619 (95% CI: 0.592-0.646) and a similar median, indicating a symmetric distribution. There is substantial variability between studies (I² ≈ 82.5%; τ = 0.273), but the prediction interval (0.481-0.742) suggests future results will still be positive. The posterior is clearly above zero, and the MCMC diagnostics (well-mixed chains, low autocorrelation, stable distributions) indicate reliable estimates. Overall, the findings support that autism interventions are effective despite differences across studies (Table 3 and Figure 4).

**Table 3:** Bayesian meta-analytic estimates for autism interventions. The pooled Bayesian effect estimate **(0.619) is consistent with the frequentist meta-analysis result (0.506 after logit transformation), indicating strong agreement between Bayesian and frequentist approaches. Dashes (-) indicate that prediction intervals are not applicable for these parameters. CI: credible interval; PI: prediction interval; MCMC: Markov Chain Monte Carlo

Parameter	Estimate	95% CI	95% PI/Range
Pooled effect (μ, mean)	0.619**	(0.592, 0.646)	(0.481, 0.742)
μ (median)	0.618	-	-
Between-study heterogeneity			
τ (SD)	0.273	(0.187, 0.383)	-
τ² (variance)	0.075	(0.035, 0.147)	-
I² (%)	82.45	(70.55, 90.95)	-
H²	6.27	(3.40, 11.05)	-
MCMC diagnostics			
μ trace range	-	-	0.25-0.75
Posterior center	≈ 0.50	-	-
Autocorrelation	-	-	Rapid decay (1.0 → ~0 by lag 2)
Chain behavior	-	-	Well-mixed, stable
Posterior shape	-	-	Unimodal, overlapping chains

Assessment of Small-Study Effects and Publication Bias

**Figure 5:**
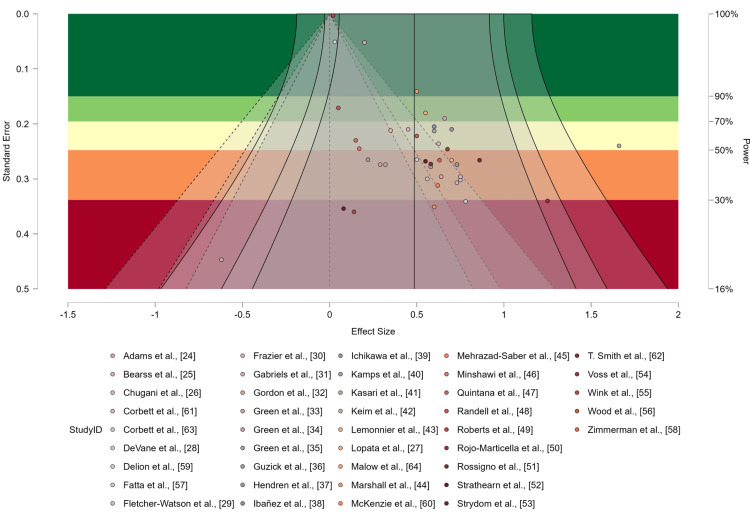
Funnel plot of included studies assessing pooled ASD interventions. Most studies fall in the low-to-moderate power range (30-50%), meaning many were underpowered. Only a few reached adequate power (≥70%), and very few exceeded 90% power. Smaller, less precise studies show more spread in effect sizes, suggesting possible small-study effects or publication bias, while larger studies cluster around more consistent, moderate effects. ASD: autism spectrum disorder Studies included [24-64].

Funnel plot asymmetry was assessed using three tests, with both the meta-regression (z = 3.429, p < 0.001) and weighted regression (t = 9.573, df = 39, p < 0.001) showing significant asymmetry, suggesting possible small-study effects or publication bias, while the rank correlation test was not significant (τ = -0.178, p = 0.103). Overall, this indicates funnel plot asymmetry, with smaller studies tending to report larger effects. The trim-and-fill analysis further supported potential publication bias by imputing 10 missing studies, which reduced the pooled effect to μ = 0.374 (95% CI (0.258, 0.491), p < 0.001), although it remained significant. Heterogeneity also remained substantial (τ = 0.338), indicating that while the corrected effect is smaller, a positive effect persists after adjustment (Table 4 and Figure 5).

**Table 4:** Funnel plot asymmetry tests. The meta-regression and weighted regression tests indicate significant asymmetry, suggesting potential small-study effects, whereas the rank correlation test was not statistically significant. Estimates μ and τ are on the logit-transformed prevalence scale. Meta-regression and weighted regression tests assess funnel plot asymmetry; non-significant p-values indicate minimal evidence of publication bias. A p-value of < 0.05 is considered significant, denoted with *. df: degrees of freedom; μ: limit estimate; CI: confidence interval

Method	Statistic	df	p-value	Estimate (μ)	95% CI	Missing Studies	Adjusted τ	95% CI (τ)
Meta-regression test	z = 3.429	-	<0.001*	0.129	(-0.079, 0.337)	-	-	-
Weighted regression test	t = 9.573	39	<0.001*	0.014	(0.005, 0.022)	-	-	-
Rank correlation test	τ = -0.178	-	0.103	-	-	-	-	-
Trim-and-fill	-	-	<0.001*	0.374	(0.258, 0.491)	10	0.338	(0.252, 0.474)

Diagnostic Evaluation of Heterogeneity and Publication Bias

**Figure 6:**
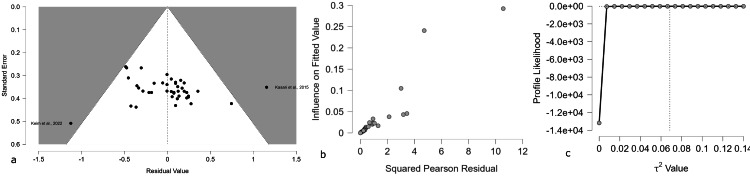
(a) Residual funnel plot illustrating the distribution of standardized residuals against their standard errors to assess small-study effects and potential publication bias; (b) Baujat plot displaying the contribution of individual studies to overall heterogeneity and their influence on the pooled effect size; and (c) profile likelihood plot for τ², depicting the estimation of between-study variance and its associated uncertainty.

To assess the robustness of the pooled effect and explore potential sources of heterogeneity, two diagnostic plots were generated. The Baujat plot showed that most studies contributed little to overall heterogeneity, but two studies exerted disproportionate influence on both variability and the pooled effect estimate [41,42]. In parallel, the funnel plot indicated that while the majority of studies were symmetrically distributed within the funnel boundaries, these two studies appeared as outliers, suggesting mild asymmetry. Taken together, these diagnostics suggest that heterogeneity was largely driven by a handful of influential studies, and although some asymmetry was present, the overall pooled effect remained robust, with deviations more consistent with genuine variability than with widespread publication bias (Figure 6).

Assessment of Funnel Plot Asymmetry and the Impact of Trim and Fill Adjustment on Effect Size Estimates

**Figure 7:**
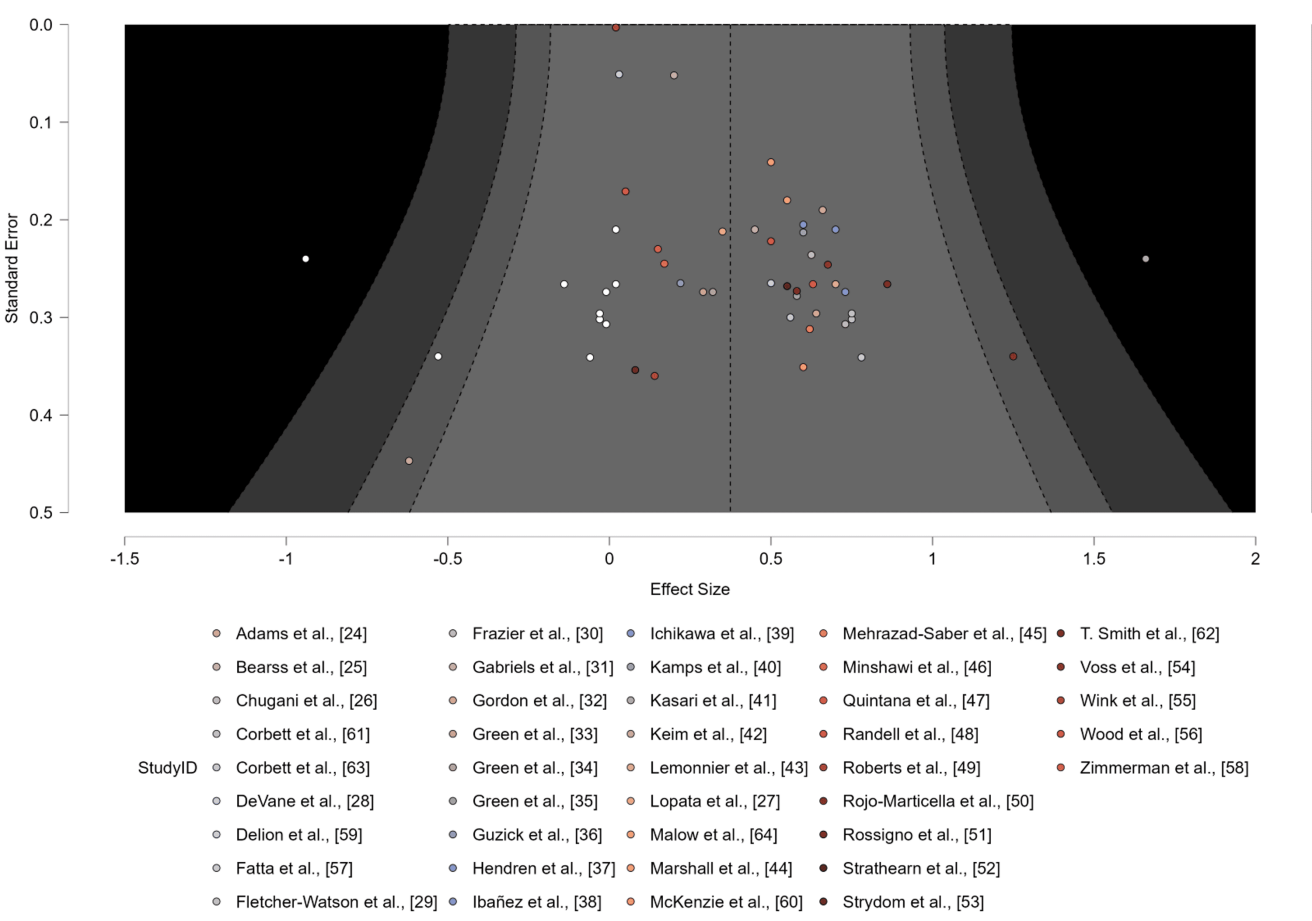
Funnel plot after the trim and fill adjustment, illustrating the correction for publication bias and a more symmetrical distribution of studies. Studies included [24-64].

The random‑effects meta‑analysis yielded a pooled logit effect of 0.506, 95% CI (0.392, 0.619), corresponding to an estimated proportion of 62% (95% CI (59%, 65%)) when back-transformed to the proportion scale. Application of the trim-and-fill method suggested 10 potentially missing studies, producing an adjusted pooled estimate of 0.374 (95% CI: 0.258-0.491, p < 0.001) for μ, and 0.338 (95% CI: 0.252-0.474, p < 0.001) for τ. The back-transformed estimate corresponds to an approximate probability of 59%, indicating a comparable magnitude of effect after adjustment. Both the original and adjusted estimates remained statistically significant, suggesting the robustness of the intervention effect even after accounting for potential publication bias. Overall, while the observed effect size may be slightly inflated, the findings indicate that ASD interventions consistently demonstrate meaningful benefits across diverse populations (Table 5 and Figure 7).

**Table 5:** Trim and fill parameter estimates for mean (μ). *A p-value of <0.05 is considered significant.

Trim and Fill Estimates	Missing	Adjusted μ	Lower 95% CI		Upper 95% CI	p
41	10	0.374		0.258	0.491	<0.001*

Sensitivity Analysis and Robustness of Findings

**Figure 8:**
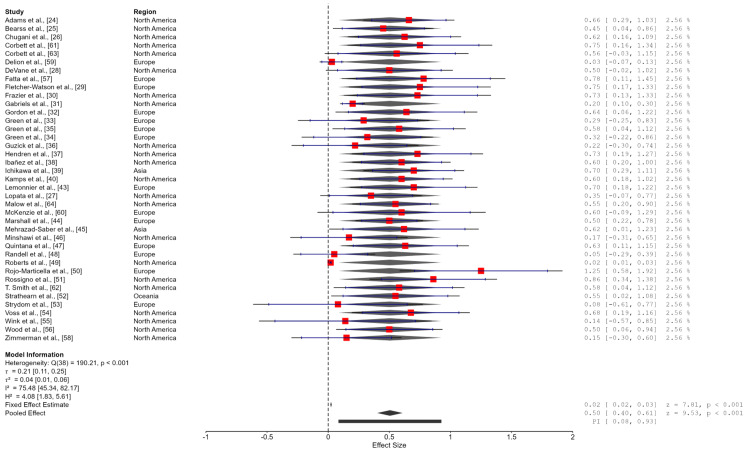
Sensitivity analysis forest Sensitivity analysis forest plot of the pooled effect size and heterogeneity of interventions across studies, with the two extreme studies [41,42] removed (2004-2025). Studies included [24-40,43-64].

After conducting a sensitivity analysis, which involved removing outlier studies identified via the residual funnel plot (Figure 6a), namely Kasari et al. [41] and Keim et al. [42], the overall pooled effect remained robust. The sensitivity analysis yielded a pooled effect estimate of 0.505 (95% CI (0.401, 0.609)), corresponding to an estimated probability of a positive outcome of approximately 62% (95% PI (0.084, 0.926). Heterogeneity among studies remained substantial, with a significant Q-test indicating variability across studies (Qₑ (38) = 190.21, p < 0.001). The between-study variance was moderate, with τ² = 0.043 (τ = 0.208), while the I² statistic of 75.49% suggests that a large proportion of observed variability is due to real differences between studies rather than sampling error. The H² value of 4.08 further confirms considerable heterogeneity. Overall, the sensitivity analysis shows that removing high-effect outliers had minimal impact on the estimated effect, indicating that the original findings were robust. Despite some reduction in between-study variance, variability in intervention effects across studies remains substantial, as reflected in the wide prediction interval. This highlights the importance of contextual factors such as participant characteristics, intervention type, and implementation setting when interpreting the overall effectiveness of ASD interventions (Table 6 and Figure 8).

**Table 6:** Sensitivity analysis of pooled ASD intervention effects (outliers removed). Outlier studies [41,42] identified via the residual funnel plot (Figure 6a) were removed. CI: confidence interval; PI: prediction interval; τ: between-study standard deviation; τ²: between-study variance; ASD: autism spectrum disorder

Statistic	Value	95% CI	95% PI
Pooled effect (logit)	0.505	0.401-0.609	0.084-0.926
τ (between-study SD)	0.208	0.108-0.255	-
τ² (between-study variance)	0.043	0.012-0.065	-
I² (%)	75.49	45.34-82.17	-
H²	4.08	1.83-5.61	-
Qₑ (df = 38)	190.21	-	-
p-value (Qₑ)	<0.001	-	-
Pooled effect z	9.53	-	-
p-value (z)	<0.001	-	-

Frequentist Subgroup Analysis

**Figure 9:**
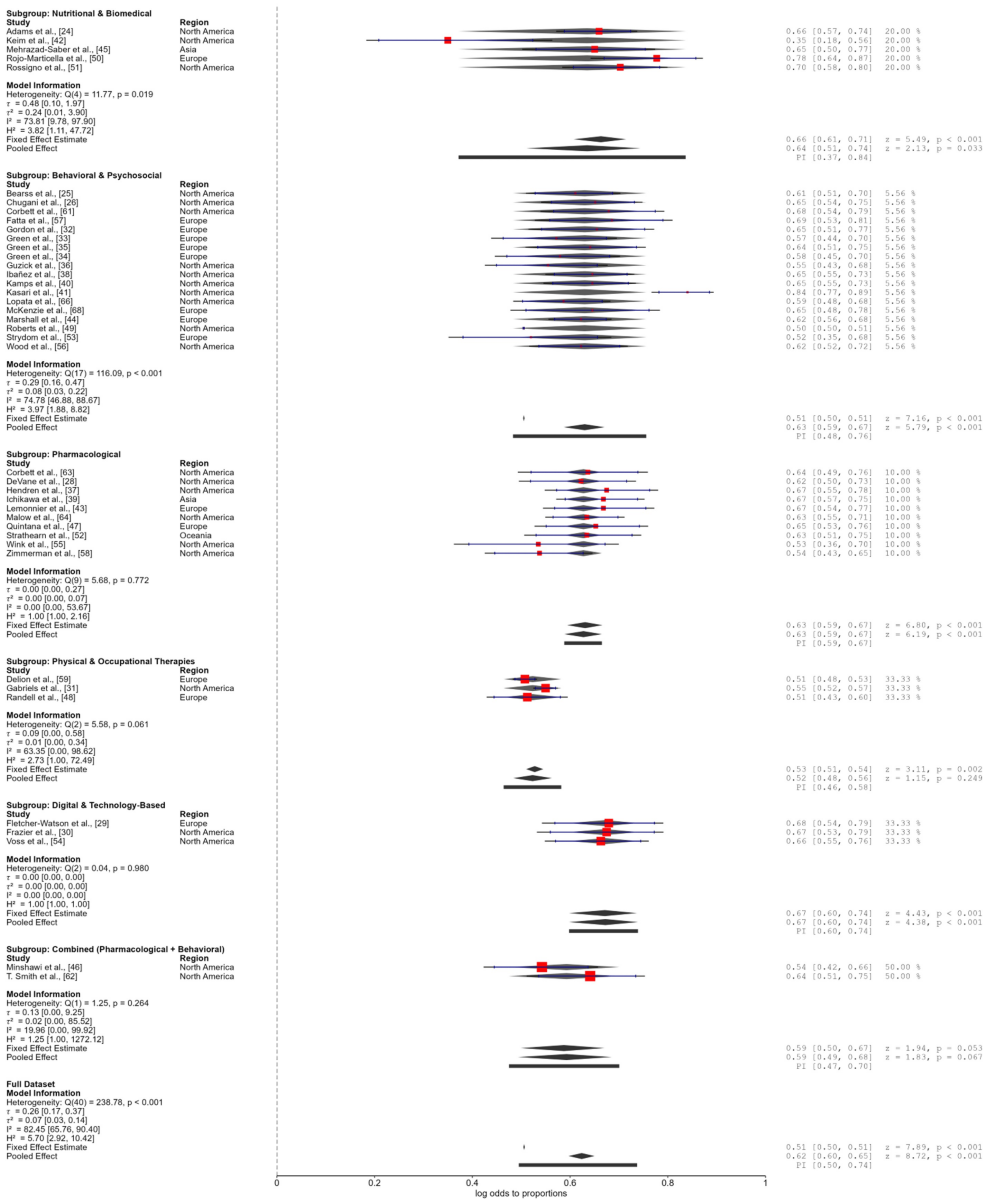
Forest plot and frequentist subgroup analysis of intervention effectiveness by intervention category (n = 41). The figure presents forest plot-based meta-analytic estimates showing pooled effect sizes for each intervention category with corresponding 95% confidence intervals and test statistics. It also illustrates within-subgroup heterogeneity, highlighting variability in effect sizes across studies. In addition, the frequentist subgroup analysis displays pooled effect estimates (proportions) across intervention subgroups, demonstrating varying degrees of intervention effectiveness across categories. Studies included [24-64].

By intervention category: The subgroup analysis showed variation in effectiveness across intervention categories. Nutritional and biomedical, behavioral and psychosocial, and pharmacological interventions all demonstrated moderate effects (64%, 63%, and 63%, respectively) (0.635, 95% CI (0.511, 0.744), PI (0.372, 0.837), p = 0.033, I² = 73.81%; 0.630, 95% CI (0.587, 0.671), PI (0.483, 0.756), p < 0.001, I² = 74.78%; 0.627, 95% CI (0.588, 0.665), p < 0.001, I² = 0%), although heterogeneity was high for nutritional and biomedical and behavioral and psychosocial interventions, while pharmacological interventions showed no heterogeneity. Digital and technology-based interventions had the highest pooled effect (67%) (0.672, 95% CI (0.598, 0.739), p < 0.001, I² = 0%), indicating a strong and highly consistent effect. In contrast, physical and occupational therapies showed a smaller, non-significant effect (52%) (0.523, 95% CI (0.484, 0.563), p = 0.249, I² = 63.35%), while combined pharmacological and behavioral interventions demonstrated a marginal effect (59%) (0.593, 95% CI (0.493, 0.685), p = 0.067, I² = 19.96%). Subgroup differences were statistically significant, Qₘ (5) = 22.63, p < 0.001, confirming variation across intervention types. Overall, although effect sizes were relatively similar across most categories, consistency varied considerably, with pharmacological and digital and technology-based interventions showing the most stable effects compared to the more heterogeneous nutritional and behavioral approaches (Table 7 and Figure 9).

**Table 7:** Subgroup meta-analytic effect estimates for intervention categories. Effect sizes are presented as proportions. PI: prediction interval. Subgroup differences were statistically significant, Qₘ (5) = 22.63, p < 0.001. A p-value of < 0.05 is considered significant, denoted with *.

Subgroup	Effect Size (Proportion)	95% CI	PI	p-value	I² (%)	Heterogeneity
Nutritional and Biomedical	0.635	(0.511, 0.744)	(0.372, 0.837)	0.033*	73.81	High
Behavioral and Psychosocial	0.630	(0.587, 0.671)	(0.483, 0.756)	<0.001*	74.78	High
Pharmacological	0.627	(0.588, 0.665)	-	<0.001*	0.00	Non
Physical and Occupational Therapies	0	(0.484, 0.563)	-	0.249	63.35	Moderate
Digital and Technology-Based	0.672	(0.598, 0.739)	-	<0.001*	0.00	Non
Combined (Pharmacological and Behavioral)	0.593	(0.493, 0.6850	(0.475, 0.701)	0.067	19.96	Low

By geographic region: The subgroup analysis examined variation in intervention effectiveness across geographic regions, including North America, Europe, and Asia. North America (62%) (0.619, 95% CI (0.582, 0.655), PI (0.475, 0.745), p < 0.001; I² = 84.21%) and Europe (63%) (0.626, 95% CI (0.579, 0.671), PI (0.495, 0.741), p < 0.001; I² = 62.34%) showed comparable moderate effects, although heterogeneity was substantial in North America and moderate in Europe. Asia (66%) (0.659, 95% CI (0.572, 0.737), p < 0.001; I² = 0%) demonstrated a similar pooled effect with no observed heterogeneity; however, this finding should be interpreted with caution due to the limited number of included studies. Subgroup differences were not statistically significant (Qₘ (2) = 0.73, p = 0.694), indicating no evidence of variation in pooled effects across regions. The Oceania subgroup could not be estimated due to insufficient data. Overall, effect sizes were consistent in magnitude across regions, with variability largely driven by within-group heterogeneity, particularly in North America and Europe (Table 8).

**Table 8:** Frequentist subgroup meta-analysis of intervention effectiveness by geographic region. Effect sizes are presented as pooled proportions with 95% confidence intervals (CIs) and prediction intervals (PIs) where available. Oceania was excluded due to insufficient studies (fewer than two estimates). Subgroup differences were not statistically significant, Qₘ (2) = 0.73, p = 0.694. A p-value of < 0.05 is considered significant, denoted with *.

Region	n (Studies)	Effect Size (Proportion)	95% CI	95% PI	p-value	I² (%)	τ²
North America	24	0.619	(0.582, 0.655)	(0.475, 0.745)	<0.001*	84.21	0.083
Europe	14	0.626	(0.579, 0.671)	(0.495, 0.741)	<0.001*	62.34	0.064
Asia	2	0.659	(0.572, 0.737)	-	<0.001*	0.00	0.000
Oceania	0	-	-	-	-	-	-

By age at start of intervention: The subgroup analysis examined variation by age at intervention onset, including children, youth, adults, children and adolescents, infants, and toddlers. Children (49%) (0.486, 95% CI (0.364, 0.609), PI (0.098, 0.874), p < 0.001; I² = 48.06%) showed a moderate effect with moderate heterogeneity. Youth (41%) (0.406, 95% CI (0.119, 0.693), p = 0.006; I² = 0%) and adults (49%) (0.487, 95% CI (0.136, 0.837), p = 0.006; I² ≈ 0%) yielded comparable effects with no observed heterogeneity, though both are based on few studies. Children and adolescents (62%) (0.620, 95% CI (0.233, 1.007), PI (-0.178, 1.418), p = 0.002; I² = 76.66%) showed a larger but more variable effect, while infants (45%) (0.450, 95% CI (0.067, 0.833), p = 0.021; I² = 0%) demonstrated a moderate effect with no heterogeneity. In contrast, toddlers (84%) (0.840, 95% CI (-0.767, 2.447), PI (-1.924, 3.604), p = 0.306; I² = 97.86%) showed a highly variable and non-significant effect. Subgroup differences were not statistically significant (Qₘ (5) = 0.98, p = 0.964), indicating no evidence of variation in pooled effects by age at intervention onset. Overall, effects were broadly comparable across age groups, though heterogeneity varied substantially, particularly among children and adolescents and toddlers, suggesting important contextual and developmental influences (Table 9).

**Table 9:** Frequentist subgroup meta-analysis of intervention effectiveness by age at intervention onset. Effect sizes are pooled proportions with 95% confidence intervals (CIs) and prediction intervals (PIs) where available. Subgroup differences were not statistically significant, Qₘ (5) = 0.97, p = 0.965. Estimates are based on log odds-to-proportion transformation. A p-value of < 0.05 is considered significant, denoted with *.

Age Group	n (Studies)	Effect Size (Proportion)	95% CI	95% PI	p-value	I² (%)	τ²
Children	25	0.617	(0.587, 0.647)	(0.522, 0.705)	<0.001*	48.60	0.036
Youth	4	0.600	(0.530, 0.6670	(0.530, 0.667)	0.006*	0.00	0.000
Adults	3	0.619	(0.534, 0.698)	(0.534, 0.698)	0.006*	0.01	0.000
Children and Adolescents	5	0.650	(0.558, 0.732)	(0.456, 0.805)	0.002*	76.66	0.127
Infants	2	0.611	(0.517, 0.697)	(0.517, 0.697)	0.021*	0.00	0.000
Toddlers	2	0.698	(0.317, 0.920)	(0.127, 0.974)	0.306	97.86	1.316

Bayesian Subgroup Meta-Analysis by Intervention Type

**Figure 10:**
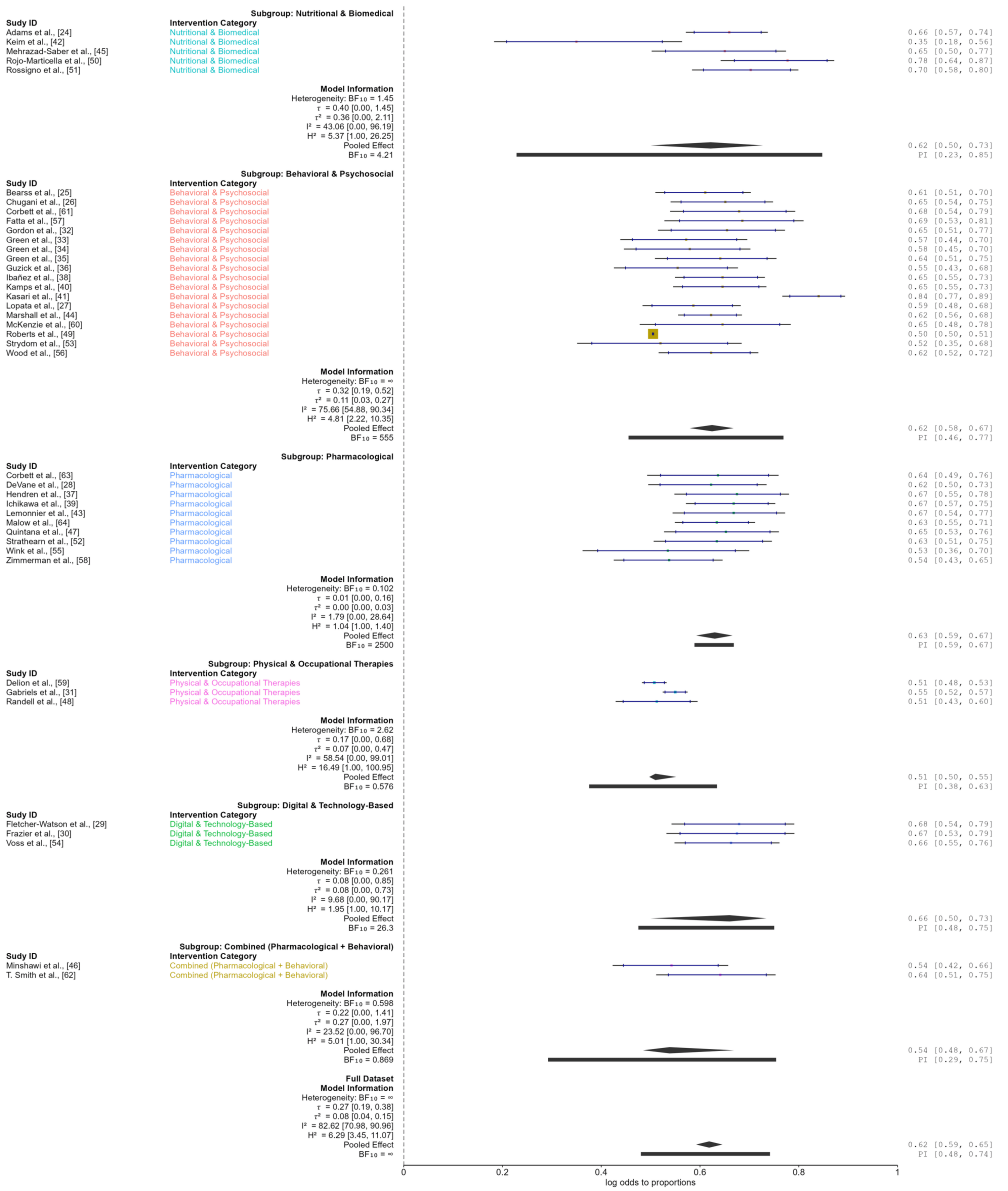
Forest plot of Bayesian subgroup meta-analysis by intervention type. Squares represent pooled posterior mean effect estimates, and horizontal lines indicate 95% credible intervals (CrIs); the vertical line denotes the null effect. Subgroups include behavioral/psychosocial, nutritional/biomedical, pharmacological, physical/occupational, digital/technology-based, and combined pharmacological-behavioral interventions. Bayes factors (BF) and heterogeneity (I²) are reported for each subgroup. Studies included [24-64].

The Bayesian subgroup meta-analysis demonstrated consistent positive pooled effects across most intervention categories, although the strength of evidence and degree of heterogeneity varied substantially between subgroups. Behavioral and psychosocial interventions showed a moderate positive effect (62.5%) 0.625 (95% credible interval CI: 0.579-0.669) with decisive evidence for inclusion (BF ≈ 832.50), but heterogeneity was substantial (I² ≈ 75.6%), indicating considerable variability across studies. Nutritional and biomedical interventions also demonstrated a moderate positive effect (62.5%) 0.625 (95% CI: 0.500-0.728), with moderate evidence (BF = 4.67) and moderate heterogeneity (I² ≈ 40.3%). Pharmacological interventions produced a comparable moderate effect (63.0%) 0.630 (95% CI: 0.592-0.665) with decisive evidence (BF = ∞) and negligible heterogeneity (I² ≈ 1.7%), indicating highly consistent findings. Physical and occupational therapies showed a smaller effect (51.0%) 0.510 (95% CI: 0.498-0.551) with weak evidence against inclusion (BF = 0.63) and moderate to high heterogeneity (I² ≈ 55.9%). Digital and technology-based interventions demonstrated one of the strongest pooled effects (66.1%) 0.661 (95% CI: 0.500-0.736) with very strong evidence (BF ≈ 30.92) and low heterogeneity (I² ≈ 9.1%), indicating relatively consistent effects. Combined pharmacological and behavioral interventions yielded a moderate effect (53.9%) 0.539 (95% CI: 0.482-0.669), but with inconclusive evidence (BF = 0.92) and low to moderate heterogeneity (I² ≈ 23.5%), suggesting some uncertainty in their overall effectiveness (Table 10 and Figure 10).

**Table 10:** Bayesian subgroup meta-analysis by intervention type. CI: credible interval; BF: Bayes factor; I²: percentage of total variation due to heterogeneity. Values are derived from Bayesian meta-analytic models using log-odds-transformed proportions.

Intervention type	Effect (Mean)	95% CI	BF	I² (%)	Interpretation
Nutritional and Biomedical	0.625	0.500-0.728	4.67	40.3	Moderate effect; moderate evidence; moderate heterogeneity
Behavioral and Psychosocial	0.625	0.579-0.669	832.50	75.6	Moderate effect; decisive evidence; substantial heterogeneity
Pharmacological	0.630	0.592-0.665	∞	1.7	Moderate effect; decisive evidence; negligible heterogeneity
Physical and Occupational Therapies	0.510	0.498-0.551	0.63	55.9	Small effect; weak evidence; moderate-high heterogeneity
Digital and Technology-Based	0.661	0.500-0.736	30.92	9.1	Strongest effect; very strong evidence; low heterogeneity
Combined (Pharmacological + Behavioral)	0.539	0.482-0.669	0.92	23.5	Moderate effect; inconclusive evidence; low-moderate heterogeneity

Meta-Regression Analyses

Meta-regression analyses indicated that intervention context (Qₘ = 18.159, df = 8, p = 0.020), outcome domain (Qₘ = 19.588, df = 6, p = 0.003), age at start (Qₘ = 17.795, df = 5, p = 0.003), and intervention category (Qₘ = 31.714, df = 5, p < 0.001) significantly moderated effect sizes, whereas follow-up duration (Qₘ = 2.679, df = 5, p = 0.749) and intervention duration (Qₘ = 1.777, df = 2, p = 0.411) did not. At the coefficient level, most contextual comparisons were associated with significantly larger effects relative to the reference category, including inpatient/clinical (B = 1.803, p = 0.002), community-based (B = 1.536, p = 0.003), school-based (B = 1.367, p = 0.003), hospital-based (B = 1.322, p = 0.041), home-based (B = 1.017, p = 0.002), and clinic-based settings (B = 1.033, p = 0.015). Outcome domain analyses showed no consistently significant individual contrasts, although adaptive functioning showed a trend toward larger effects (B = 0.659, p = 0.059). Age at start significantly moderated outcomes, with toddlers demonstrating larger effects (B = 1.326, p = 0.012), while other age groups were not significant. Intervention category also significantly moderated effects, with physical and occupational therapies (B = -1.230, p < 0.001) and combined pharmacological and behavioral interventions (B = -0.792, p = 0.009) associated with smaller effect sizes relative to the reference category. Overall, these findings suggest that heterogeneity in effect sizes is primarily explained by intervention context, age, and intervention type, whereas timing-related variables such as follow-up and duration did not significantly contribute to variability (Table 11).

**Table 11:** Meta-regression results examining moderators of intervention effect sizes. Qₘ: omnibus test statistic for moderator effects in meta-regression; df: degrees of freedom; p: p-value; fixed-effects meta-regression tests were conducted using a z-distribution. A p-value of < 0.05 is considered significant, denoted with *.

Predictor	Qₘ	df	p-value
Context	18.159, p = 0.020*	8	0.020
Follow-Up	2.679, p = 0.749	5	0.749
Outcome Domain	19.588, p = 0.003*	6	0.003*
Age at Start	17.795, p = 0.003*	5	0.003*
Intervention Duration	1.777, p = 0.411	2	0.411
Intervention Category	31.714, p < 0.001*	5	<0.001*

Multicollinearity among moderators was assessed using variance inflation diagnostics. Because several moderators were categorical variables represented by multiple parameters, scaled inflation factors (SIFs) were examined alongside VIFs. SIF values ranged from 1.99 to 6.33, indicating generally low levels of multicollinearity and no evidence of severe collinearity among moderators included in the meta-regression models. Although intervention duration exhibited a moderately elevated SIF (6.33), it remained below commonly accepted thresholds for serious multicollinearity (Appendix C).

Discussion

Principal Findings

The observed heterogeneity was likely attributable to multiple factors, including differences in intervention modalities, delivery settings, participant characteristics, outcome domains, and study design features. Diagnostic and analytical variability, as well as differences in implementation fidelity and follow-up duration, may have further contributed to the observed dispersion in effect sizes. Thus, while the overall evidence supports the effectiveness of ASD interventions, variability in outcomes - rather than the pooled estimate alone - emerges as a central finding of this synthesis, underscoring the context-dependent nature of intervention success, pinpointing the need for careful consideration of population characteristics, intervention design, and delivery settings in both research and practice. Importantly, beyond statistical significance, the pooled effect size (0.506; 95% CI: 0.392-0.619) indicates a moderate and potentially clinically meaningful benefit of ASD interventions, corresponding to an estimated success proportion of approximately 62%. This suggests that ASD interventions can produce measurable improvements in developmental, behavioral, and functional outcomes that may translate into gains in daily functioning, educational participation, caregiver well-being, and quality of life. The practical relevance of these findings is further supported by the consistency of results across frequentist, Bayesian, and sensitivity analyses, although the substantial heterogeneity observed indicates that intervention effectiveness remains highly dependent on population, intervention, and implementation context. This interpretation is further supported by the meta-regression analyses, which provide a formal test of whether study-level characteristics account for the observed heterogeneity. However, the meta-regression results indicated that heterogeneity was primarily explained by intervention context, age at intervention start, outcome domain, and intervention category, whereas follow-up duration and intervention duration were not statistically significant moderators, suggesting that not all study-level characteristics contributed equally to observed variability. Findings should be interpreted cautiously and considered exploratory. Additional high-quality studies are needed to provide more precise estimates of effectiveness for these intervention types.

Variation by Intervention Type and Population Context

Subgroup analyses showed broadly similar effectiveness across intervention types, with improvement rates of about 52% to 67%. Digital approaches performed best, while physical and occupational therapies were lowest. Although effect sizes were similar, variability remained high in several groups (I² = 63-75%). Pharmacological and digital interventions showed almost no variability, suggesting more consistent results. Regional outcomes were also similar (62-66%) with no major differences, though some variation still existed within regions. Age-based analyses followed the same pattern, but variability was higher, especially among toddlers. Overall, results were consistent across subgroups, but variation within them remained notable, pointing to the influence of context. These subgroup patterns are broadly consistent with the meta-regression findings, which identified intervention category as a significant source of heterogeneity, particularly highlighting reduced effects for physical and occupational therapies relative to other intervention types.

Methodological and Contextual Moderators

Meta-regression identified key factors influencing outcomes, including setting, duration, outcome type, age, and intervention type. Omnibus tests indicated that context, outcome domain, age at start, and intervention category significantly explained heterogeneity (context: Qₘ = 18.159, p = 0.020; outcome domain: Qₘ = 19.588, p = 0.003; age at start: Qₘ = 17.795, p = 0.003; intervention category: Qₘ = 31.714, p < 0.001), whereas follow-up duration and intervention duration were not significant moderators. Home-based and medium-duration (three to six months) interventions showed larger effects, while clinic- and institution-based settings showed more variable and often smaller effects. Across contexts, most settings (e.g., inpatient/clinical, community-based, school-based) were associated with significantly higher effect sizes relative to the reference category, suggesting that intervention setting plays a key role in outcome magnitude. Family/psychosocial and adaptive outcomes, as well as interventions started in infancy, toddlerhood, and youth, showed lower effects. Age at intervention start was a significant moderator, with toddlers showing notably larger effects compared to the reference age group, while other age groups were not significant. Differences between intervention types also contributed to variations in results, with physical and occupational therapies and combined pharmacological-behavioral interventions associated with significantly smaller effect sizes, whereas other categories showed non-significant differences. However, interpretation of these coefficients should be made with caution, as variance inflation diagnostics indicated substantial multicollinearity among predictors, suggesting that study-level characteristics were highly interrelated and limiting the ability to fully disentangle independent moderator effects. Consequently, observed associations may reflect overlapping contextual influences rather than isolated causal effects of individual moderators. Overall, effectiveness is strongly influenced by context and study design, with considerable unexplained variability still remaining.

Sensitivity and Robustness

Sensitivity analyses showed that a few studies contributed heavily to variability, but removing them had little effect on the overall estimate (62%). Trim-and-fill analysis suggested some publication bias, lowering the estimate slightly to 59%, though results remained significant. Overall, the findings are robust, and the variability likely reflects real differences rather than just outliers. Importantly, the persistence of significant moderator effects in the meta-regression despite these adjustments suggests that observed heterogeneity is not solely attributable to outliers or publication bias but reflects genuine variation across study contexts and intervention characteristics.

Bayesian Interpretation

Bayesian analyses closely matched frequentist results (~62-63%) and showed strong evidence of a positive effect. Although variability remained high, prediction intervals suggested that future studies are likely to show meaningful benefits. Convergence checks confirmed stable estimates. Overall, the Bayesian results support the effectiveness of ASD interventions while highlighting differences across contexts. Although Bayesian subgroup analyses were not formally modelled within the same multivariable framework, the consistency between Bayesian and frequentist subgroup patterns provides converging evidence that intervention category and contextual factors are key sources of variation in intervention effects.

Comparison With Prior Literature

Prior meta-analyses of interventions for ASD have consistently reported moderate positive effects across a range of therapeutic approaches, broadly aligning with the present pooled estimate indicating that approximately 62% of participants benefit from intervention. For example, earlier syntheses of behavioral and developmental interventions have reported effect sizes in the small-to-moderate range, particularly for early intensive behavioral interventions and parent-mediated approaches [65-67]. Similarly, umbrella reviews and large-scale evidence syntheses have concluded that, while many ASD interventions demonstrate efficacy, the magnitude of benefit is often variable and context-dependent [68-69]. The present findings extend this literature by integrating both frequentist and Bayesian approaches across a larger and more methodologically diverse evidence base, providing convergent evidence of a moderate-to-large overall effect.

Consistent with prior research, the current analysis identified substantial between-study heterogeneity (I² ≈ 75-82%), reinforcing longstanding concerns regarding variability in intervention outcomes across studies [70]. Previous meta-analyses have attributed such heterogeneity to differences in intervention intensity, fidelity, participant characteristics, and outcome measurement, particularly in complex, multi-component interventions [65,71]. The wide prediction intervals observed in the present study (~50-73%) further support the interpretation that intervention effectiveness is not uniform but varies meaningfully across contexts. This aligns with earlier work emphasizing that pooled estimates in ASD intervention research should be interpreted as average effects across heterogeneous conditions rather than universally applicable benchmarks [72,73].

Subgroup findings in the current study are also broadly consistent with prior literature. Behavioral and psychosocial interventions demonstrated moderate effectiveness, in line with established evidence supporting ABA and related approaches [66,74]. Pharmacological interventions showed comparable average effects but lower heterogeneity, reflecting findings from systematic reviews indicating more standardized protocols and outcome measures in medication trials [75-78]. Notably, digital and technology-based interventions demonstrated relatively strong and consistent effects, supporting emerging evidence that technology-assisted therapies may enhance engagement and scalability, although this remains an evolving area of research [79,80]. In contrast, physical and occupational therapies showed smaller and more variable effects, consistent with previous reviews highlighting mixed evidence and methodological limitations in these domains [81,82].

The identification of contextual and methodological moderators in the present analysis further aligns with prior findings. Previous studies have shown that intervention setting, duration, and delivery format significantly influence outcomes, with home-based and parent-mediated interventions often yielding stronger or more generalizable effects [41,83,84]. The present results similarly indicate that home-based and medium-duration interventions are associated with greater effectiveness, while clinic- and institution-based programs tend to yield smaller effects. Moreover, variability across outcome domains observed here reflects ongoing challenges in measuring complex constructs such as adaptive functioning and psychosocial outcomes, which have been noted as less responsive or more difficult to standardize in prior research [67,85,86].

The robustness of the present findings, demonstrated through sensitivity analyses and trim-and-fill adjustments, is also consistent with earlier meta-analyses showing that while publication bias and small-study effects may modestly inflate effect sizes, they rarely negate the overall conclusion of intervention benefit [87-89]. The close agreement between frequentist and Bayesian estimates in the current study further strengthens confidence in the results, echoing recent methodological work advocating for Bayesian approaches to better characterize uncertainty and heterogeneity in ASD research [90-92].

## Conclusions

Overall, the findings of this meta-analysis are consistent with previous research. The results indicate moderate effectiveness, with considerable variation across studies. This variation appears to be influenced by contextual, demographic, and intervention-specific factors, as confirmed by the meta-regression findings. Together, these results suggest that outcomes of ASD interventions are not determined by a single superior approach, but rather by how, where, and for whom the intervention is implemented. Therefore, context-sensitive and individualized intervention strategies are essential. However, given evidence of substantial multi-collinearity among moderator variables, these findings should be interpreted as reflecting overlapping and interdependent contextual influences rather than fully independent effects of individual study-level characteristics.

## Appendices

Appendix A 

**Table 12:** Full electronic search strategy.

Database	Search Strategy	Filters/Limits	Search Date
PubMed/MEDLINE	("Autism Spectrum Disorder"[MeSH] OR "Autistic Disorder"[MeSH] OR "autism spectrum disorder" OR ASD OR autism) AND ("Intervention Studies"[MeSH] OR intervention* OR therap* OR treatment* OR program* OR rehabilitation) AND (behavioral OR developmental OR educational OR pharmacological OR medication* OR "Applied Behavior Analysis" OR ABA OR NDBI OR occupational therap* OR speech therap* OR technology-based OR digital) AND (randomized OR randomised OR "Randomized Controlled Trial"[Publication Type] OR RCT)	Humans; 2004-2025	April 30, 2025
Embase	('autism spectrum disorder'/exp OR 'autistic disorder'/exp OR autism OR ASD) AND ('intervention study'/exp OR intervention* OR therap* OR treatment* OR rehabilitation) AND ('behavior therapy'/exp OR behavioral OR developmental OR educational OR pharmacological OR medication* OR ABA OR NDBI OR occupational therapy OR speech therapy OR digital intervention) AND ('randomized controlled trial'/exp OR randomized OR randomised OR RCT)	Humans; 2004-2025	April 30, 2025
Web of Science	TS=("autism spectrum disorder" OR autism OR ASD) AND TS=(intervention* OR therap* OR treatment* OR rehabilitation OR program*) AND TS=(behavioral OR developmental OR educational OR pharmacological OR medication* OR ABA OR NDBI OR occupational therapy OR speech therapy OR digital OR technology-based) AND TS=(randomized OR randomised OR RCT OR trial)	2004-2025	April 30, 2025
Scopus	TITLE-ABS-KEY("autism spectrum disorder" OR autism OR ASD) AND TITLE-ABS-KEY(intervention* OR therap* OR treatment* OR rehabilitation OR program*) AND TITLE-ABS-KEY(behavioral OR developmental OR educational OR pharmacological OR medication* OR ABA OR NDBI OR occupational therapy OR speech therapy OR digital OR technology-based) AND TITLE-ABS-KEY(randomized OR randomised OR RCT OR trial)	2004-2025	April 30, 2025

Appendix B 

**Table 13:** Risk of bias assessment using the RoB 2 tool (supplementary to Figure 2 showing RoB 2 results).

Study ID	Randomization Process	Deviations From Intended Interventions	Missing Outcome Data	Measurement of Outcome	Selection of Reported Result	Overall RoB
Adams et al., 2018 [24]	Some concerns	Some concerns	Low	Some concerns	Some concerns	Some concerns
Bearss et al., 2015 [25]	Low	Some concerns	Low	Some concerns	Some concerns	Some concerns
Chugani et al., 2016 [26]	Low	Low	Low	Some concerns	Low	Low
Lopata et al., 2025 [27]	Some concerns	Low	Some concerns	Low	Low	Some concerns
DeVane et al., 2019 [28]	Low	Low	Low	Some concerns	Some concerns	Low
Fletcher-Watson et al., 2016 [29]	Low	Low	Low	Low	Some concerns	Low
Frazier et al., 2017 [30]	Low	Low	Low	Low	Some concerns	Low
Gabriels et al., 2015 [31]	Some concerns	Low	Low	Some concerns	Some concerns	Some concerns
Gordon et al., 2015 [32]	Low	Some concerns	Low	Some concerns	Some concerns	Some concerns
Green et al., 2010 [33]	Low	Some concerns	Low	Some concerns	Low	Some concerns
Green et al., 2017 [34]	Low	Low	Low	Low	Low	Low
Green et al., 2015 [35]	Low	Low	Low	Low	Low	Low
Guzick et al., 2024 [36]	Low	Some concerns	Low	Some concerns	Low	Some concerns
Hendren et al., 2016 [37]	Low	Low	Low	Some concerns	Low	Low
Ibañez et al., 2018 [38]	Low	Some concerns	Low	Some concerns	Some concerns	Some concerns
Ichikawa et al., 2017 [39]	Low	Low	Low	Low	Some concerns	Low
Kamps et al., 2015 [40]	Low	High	Low	Some concerns	Some concerns	Some concerns
Kasari et al., 2015 [41]	Low	Some concerns	Low	Some concerns	Low	Some concerns
Keim et al., 2022 [42]	Low	Low	Low	Low	Low	Low
Lemonnier et al., 2012 [43]	Low	Low	Low	Low	Some concerns	Low
Marshall et al., 2016 [44]	Low	Low	Low	Some concerns	Low	Some concerns
Mehrazad-Saber et al., 2018 [45]	Low	Low	Some concerns	Some concerns	Some concerns	Some concerns
Minshawi et al., 2016 [46]	Low	Low	Low	Some concerns	Some concerns	Some concerns
Quintana et al., 2017 [47]	Low	Low	Low	Low	Low	Low
Randell et al., 2022 [48]	Low	Low	Some concerns	Some concerns	Low	Some concerns
Roberts et al., 2023 [49]	Low	Low	Some concerns	Some concerns	Low	Some concerns
Rojo-Marticella et al., 2025 [50]	Low	Some concerns	Low	Some concerns	Some concerns	Some concerns
Rossigno et al., 2009 [51]	Low	Low	Low	Some concerns	Some concerns	Some concerns
Strathearn et al., 2018 [52]	Low	Low	Low	Low	Some concerns	Low
Strydom et al., 2020 [53]	Low	Low	Low	Some concerns	Some concerns	Low
Voss et al., 2019 [54]	Low	Low	Low	Some concerns	Some concerns	Low
Wink et al., 2016 [55]	Low	Low	Some concerns	Low	Some concerns	Some concerns
Wood et al., 2020 [56]	Low	Low	Some concerns	Some concerns	Low	Some concerns
Fatta et al., 2024 [57]	Low	Low	Some concerns	Some concerns	Some concerns	Some concerns
Zimmerman et al., 2021 [58]	Low	Low	Low	High	Some concerns	Some concerns
Delion et al., 2018 [59]	Low	Low	Some concerns	Some concerns	Low	Some concerns
McKenzie et al., 2019 [60]	Some concerns	Some concerns	Low	Some concerns	Low	Some concerns
Corbett et al., 2019 [61]	Low	Some concerns	Some concerns	Low	Low	Some concerns
T. Smith et al., 2016 [62]	Some concerns	Some concerns	Low	Some concerns	Low	Some concerns
Corbett et al., 2023 [63]	Some concerns	Some concerns	Some concerns	Low	Low	Some concerns
Malow et al., 2021 [64]	Low	Low	Some concerns	Some concerns	Low	Low

Appendix C 

**Table 14:** Collinearity assessment of moderators included in the meta-regression models. VIF: variance inflation factor; SIF: scaled inflation factor

Effect Size Meta-Regression Variance Inflation			
	Parameters	VIF	SIF
Intervention Category	5	4841.98	2.336
Outcome Domain	7	15156.07	1.989
Follow-Up	5	10624.14	2.527
Context	8	933128.75	2.361
Age at Start	5	64075.79	3.025
Intervention Duration (Months)	1	40.10	6.333
